# The North-Eastern Hospital for Children

**Published:** 1893-05-06

**Authors:** 


### THE INSTITUTIONAL WORKSHOP.

### WITHIN THE HOSPITALS.

### THE NORTH-EASTERN HOSPITAL FOR CHILDREN.

Anyone who pays a visit to this institution in Goldsmith's Row will be struck with the somewhat forlorn aspect which it presents, owing to an absence of funds to enable the committee to complete the buildings so as to make them front the Hackney Road. Although the hospital has been established for a quarter of a century, it seems, so far, to have failed to secure its fair share of legacies and other contributions from the benevolent. It is, we believe, almost unique amongst metropolitan hospitals in the circumstance that, at the present time, it has practically no invested funds of any kind. This statement shows the enormous financial difficulties which the Committee and their able Secretary, Mr. Alfred Nixon, have had to face. Situated in one of the poorest districts of the metropolis, upon a site which is by no means prominent, the hospital appears so far to have failed to attract the support of the wealthy, although there is no institution which deserves that support more. Legacies have been surprisingly few, and, when compared with other similar hospitals, they seem to be altogether out of proportion to the needs and deserts of this most deserving of institutions. We therefore beg to draw the attention of all philanthropic people to the neglect of this hospital by the benevolent, in the hope that they may be induced to pay an early visit to its wards, when we make no doubt that their purse-strings will be loosened, and that money will be gladly given, seeing that the evidence of good management is conspicuously present in every department of the hospital. The East London Hospital for Children, although its managers have been enabled to complete the buildings, and recently to erect a new out-patient department at a cost of upwards of £6,000, seems also to have suffered from an absence of legacies. They have, however, accumulated invested property to the extent of nearly £8,000. On the other hand, the North-Eastern Hospital appears to have no invested property whatever, a circumstance which should commend it to the liberal support of that large body of persons who are continuously insisting that every charitable institution should have the courage to depend entirely upon the support which it can secure from the public during each year of its existence. In any case, an examination of the accounts of the North-Eastern Hospital for Children, and an intelligent inquiry into its administration, impels us to declare that it deserves, as it most urgently needs, at least double the amount of monetary help which it at present receives. If we could only induce our readers to pay a visit to the wards, get them to talk to the children, and observe the devotion and efficiency of the nursing staff, we make no doubt that very many of the present governors would double their subscriptions, whilst the list of governors would be very materially increased.

#### THE ADMINISTRATION.

The wards, although erected on the pavilion principle, have linked cots, and here and there three or four cots together between the windows in the large wards. Although the nursing staff is much smaller here than at Great Ormond Street, it is noticeable that the work is exceptionally well done, and that the condition of the children leaves little, if anything, to be desired. We were very much struck by the cheerfulness of the children, and their appearance. They were essentially happy children, and every one of them seemed to welcome the approach of the nurses, who had evidently won the confidence of every child committed to their care. The nurses were not only efficient and capable, but cheerful and bright. This was the more remarkable, because, owing to the number of infants to be met with in the large wards, there can be no doubt that, according to modern ideas, the number of nurses to the children is too few. This is acknowledged by the authorities, but they find it impossible to increase the number owing to an absence of sleeping accommodation, due to a paucity of funds. It is therefore satisfactory to know that, despite the heavy character of the work, the nurses are attached to the institution, and many of them have been for a great number of years in the service. The wards contained flowers and toys, but our inquiries led us to conclude that it would be a great kindness on the part of a few ladies if they would make it their business to send a present of toys, say, once a month, and to arrange for a weekly supply of flowers for the wards. We should also like to suggest that some friend or friends of the hospital should take an early opportunity of presenting each of the wards with a large musical box.

#### SPECIAL COTS AND FURNITURE.

We noticed that, although there were a few presentation and memorial cots in the wards of this hospital, there is evidence that much might be done to increase the funds of the institution, if the Sunday and other schools of the district could de induced to undertake to support so many of the cots in each ward. We believe that if the claims of the hospital could be brought home to the teachers of the schools throughout the district, they would be willing to bring the matter to the notice of their schools, and in this way many hundreds a year might be added to the income of the hospital. The people in the immediate neighbourhood evidently take a warm interest in the well being of the North-Eastern Hospital. This is shown by the circumstance that a local committee was formed at the end of the year 1892, which raised sufficient money to present to each ward a large rocking-horse, in addition to a gift of money. These rocking-horses are interesting, from the fact that they are the gift of the local residents, and that each rocking-horse was made in the district. Whilst urging the importance of securing local support, we do no not fail to recognise the necessity of arousing and fixing the attention of wealthy residents in other parts of London to the needs of this most deserving hospital, and we hope that this article may do something in that direction, and that the committee may soon be placed in possession of sufficient funds to enable them to complete the buildings, and so to take in very many of the poor children, who at the present time have, we understand, to be refused admission for want of room.

The Out-patient Department and Appliances.

The out-patient department, though erected fifteen years ago, is fairly adequate. It is well ventilated, and although it is no doubt crowded during several days in each week, owing to the ever-increasing number of patients who flock here for relief, still the work seems to be expeditiously and thoroughly done. On the walls of the out-patient department appear frequent notices to the effect "Beware of Pickpockets." These notices have been put up because several mothers have lost their purses whilst attending the department, a circumstance which would seem to reflect somewhat unfavourably upon the inhabitants of the district. We have inspected many hundreds of out-patient departments, but we never before met with such a notice. The dispensing is undertaken by lady dispensers, and this hospital claims to be one of the first, if not the first institution, which employed ladies in the dispensary. The baths and fittings throughout the hospital will soon need to be replaced by those of a newer pattern. There is an excellent linen-room, which is artificially heated, and the cupboards in which are so arranged as to secure thorough ventilation, not only of the cupboards, but of the room itself. Having inspected an unventilated linen-room on the same day as that of our visit to this hospital, the excellent plan upon which the linen-room at the North-Eastern Hospital was constructed was more than usually apparent. Everywhere throughout the hospital there were signs of scrupulous economy, combined with an honest attempt to afford the maximum of comfort to each of the patients. The staircase at this hospital is one which deserves the attention of architects; the treads are constructed of freestone, whilst each of the risers is lined with blue encaustic tiles. The landings also are laid with encaustic tiles, and the general effect is both pleasing and excellent. Although we found it a little difficult—owing, apparently, to the lateness of our visit, five p.m.—to get over the hospital, we left iit with the feeling that it was most efficiently admin stered, and that it deserved a much larger measure of pecuniary support than it at present receives. Much of its present efficiency is evidently due to Miss Gray, the housekeeper, who courteously showed us over the building, and who has been in the service of the institution for upwards of thirteen years. Miss Gray would make a model matron for any convalescent institution, a fact which we mention for the information of the committees of these charities, knowing from experience how difficult it often is to meet with a lady possessing the requisite experience and gifts to render her efficient for such an important office.

